# Different Effects of BORIS/CTCFL on Stemness Gene Expression, Sphere Formation and Cell Survival in Epithelial Cancer Stem Cells

**DOI:** 10.1371/journal.pone.0132977

**Published:** 2015-07-17

**Authors:** Loredana Alberti, Lorena Losi, Serge Leyvraz, Jean Benhattar

**Affiliations:** 1 Institute of Pathology, Lausanne University Hospital, Lausanne, Switzerland; 2 Department of Life Sciences, University of Modena and Reggio Emilia, Modena, Italy; 3 Department of Oncology, Lausanne University Hospital, Lausanne, Switzerland; 4 Biopath Lab, Lausanne, Switzerland; University of Newcastle, UNITED KINGDOM

## Abstract

Cancer stem cells are cancer cells characterized by stem cell properties and represent a small population of tumor cells that drives tumor development, progression, metastasis and drug resistance. To date, the molecular mechanisms that generate and regulate cancer stem cells are not well defined. BORIS (Brother of Regulator of Imprinted Sites) or CTCFL (CTCF-like) is a DNA-binding protein that is expressed in normal tissues only in germ cells and is re-activated in tumors. Recent evidences have highlighted the correlation of *BORIS/CTCFL* expression with poor overall survival of different cancer patients. We have previously shown an association of BORIS-expressing cells with stemness gene expression in embryonic cancer cells. Here, we studied the role of BORIS in epithelial tumor cells. Using BORIS-molecular beacon that was already validated, we were able to show the presence of *BORIS* mRNA in cancer stem cell-enriched populations (side population and spheres) of cervical, colon and breast tumor cells. BORIS silencing studies showed a decrease of sphere formation capacity in breast and colon tumor cells. Importantly, BORIS-silencing led to down-regulation of *hTERT*, stem cell (*NANOG*, *OCT4*, *SOX2* and *BMI1*) and cancer stem cell markers (*ABCG2*, *CD44* and *ALDH1*) genes. Conversely, BORIS-induction led to up-regulation of the same genes. These phenotypes were observed in cervical, colon and invasive breast tumor cells. However, a completely different behavior was observed in the non-invasive breast tumor cells (MCF7). Indeed, these cells acquired an epithelial mesenchymal transition phenotype after BORIS silencing. Our results demonstrate that BORIS is associated with cancer stem cell-enriched populations of several epithelial tumor cells and the different phenotypes depend on the origin of tumor cells.

## Introduction

Enormous evidences support the view that human cancer could be considered as a stem cell disease [1–3]. The cancer stem cells (CSCs) theory assumes that cancers are viewed as complex tissues where aberrant cell growth is driven by a small population of cells defined as tumor-initiating cells. These cells are characterized by three main properties: uncontrolled proliferation capacity, ability of self-renewal and ability to differentiate into a non-CSC progeny [3, 4]. Interestingly, the properties of these malignant cells are closely similar to the three features that characterize normal stem cells: the potential to proliferate extensively, the self-renewal and the capability to develop into multiple lineages. Accordingly, tumor cells with these properties are also called cancer stem cells. The first observations of the presence of CSCs were performed in human acute myeloid leukemia [5] and consequently developed in different types of human solid tumors, as breast [6], brain [7], colon [8, 9], pancreatic [10] and ovarian [11] tumors. It has been shown that many cancer patients, especially with solid tumors, respond poorly to conventional therapies, such as chemotherapy and radiotherapy, and after an initial partial remission, tumors relapse. The reasons of such failure could be explained by the drug- and radio- resistance of CSCs. In addition, it has been demonstrated that CSCs are more frequent in highly aggressive and refractory tumors [6–7]. Therefore, it becomes extremely important to identify the CSC populations and their markers to develop CSC-targeted therapies to overcome the resistance of CSCs to the conventional anti-cancer drugs. Using experimental approaches, the CSCs of many tumor types have been characterized phenotypically and several CSC markers have been identified [4, 12]. However, most of the identified markers are not fully specific to CSCs because they are also expressed in normal cells, and therefore, the use of multiple markers is required. Many efforts on cancer research will be necessary to optimize targeting CSCs therapies. There are several approaches to enrich the CSCs populations, which are mainly used for *in vitro* analyses and screening methods. One approach is based on the selection of a cell subpopulation that is able to efflux dyes. The efflux of these dyes is a capacity of CSCs which express genes encoding the ATP-binding cassette (ABC) drug transporters, such as ABCG2 [13–15]. The most used dye is Hoechst 33342, which is a DNA-binding dye. The subpopulation selected by this method is called side population (SP). The aldehyde dehydrogenase (ALDH) activity is another functional property of stem cells, used to isolate enriched CSCs population [16, 17]. An additional *in vitro* approach is based on non-adherent serum-free culture [8, 18]. Using this method, the cells from different type of tumors (including brain, breast and colon), which have the capacity of self-renewal and to maintain stem-cell properties, can form spheroid colonies named spheres [19].

BORIS (Brother of Regulator of Imprinted Sites) is a DNA-binding protein which shares with its paralog CTCF, an 11 zinc-finger domain, thus also called CTCFL (CTCF-like) [20]. BORIS protein is involved in epigenetic reprogramming and it belongs to cancer testis antigen family, as it is expressed in normal germinal cells and reactivated in tumors. Recent reports indicate that BORIS expression is associated with advanced stage in different cancers, such as ovarian, prostate, esophageal and hepatocellular cancers [21–24]. In ovarian cancers, BORIS expression may also confer poor prognosis [21]. Our previous study has demonstrated the association of BORIS expression with stem cell and CSC marker genes in embryonic carcinoma cells [25]. Altogether these evidences prompted us to further investigate the presence and the molecular functions of BORIS in the CSCs-enriched populations in other types of tumor cells and specifically in cervical, colon and breast tumor cells. As there is not yet a validated antibody against BORIS, we used the BORIS-molecular beacon (BORIS-MB) that was previously tested and validated for *in vivo* detection of *BORIS* mRNA [25]. BORIS-MB allowed us to visualize the BORIS-positive cells in the analyzed epithelial tumor cells. Interestingly, we found that *BORIS* is highly expressed in CSC-enriched populations isolated from SP and spheres. Furthermore, functional studies revealed that BORIS could play an important role in the self-renewal of tumors and in the acquisition of epithelial mesenchymal transition (EMT) signature in base of the origin of the tumor cells.

## Materials and Methods

### Cells and spheres preparation

The human cell lines (HeLa, cervical adenocarcinoma; HT29, colon adenocarcinoma; NCCIT, embryonic carcinoma) were purchased from the American Type Culture Collection (ATCC) and the human breast cell lines (MCF7 and MDA-MB-231) were provided by Dr Stéphanie Renaud (Biotechnology Institute, University of Lausanne). The cells were cultured at 37°C with 5% CO_2_ either in Dulbecco's modified Eagle's medium (DMEM; Gibco, Invitrogen) for HeLa and HT29 cells, or in RPMI-1640 medium (Gibco, Invitrogen) for NCCIT, MCF7 and MDA-MB-231 cells, supplemented with 10% of heat inactivated fetal bovine serum (FBS; Invitrogen) and 1% of Penicillin-Streptomycin (Gibco, Invitrogen).

For sphere culture, cells (HT29, MCF7 and MDA-MB-231) were first detached with 0.25% trypsin solution (Invitrogen) and washed twice in PBS (Invitrogen). Then, cells were filtrated twice using a cell-strainer of 40 μm mesh size (Falcon) and cultured in serum-free medium containing DMEM/F-12 medium (Invitrogen) supplemented with B27 (Invitrogen), 5 μg/ml heparin (Sigma), 20 ng/ml EGF (Epidermal Growth Factor, BD Biosciences), 20 ng/ml FGF (Fibroblast Growth Factor, BD Biosciences) and 5 μg/ml insulin (Sigma). Cells were plated into ultra-low attachment 6-well plates (Corning) at the density of 1,000 cells/ml for 10–15 days. Spheres were counted and collected for RNA extraction. An aliquot of spheres was seeded in normal medium with serum to allow the differentiation.

### Fluorescence analysis using BORIS-MB

Cells were prepared as previously described [25]. Briefly, cells in suspension (1 x 10^6^ cells/ml) were incubated at 37°C for 1.5 hour in serum-free DMEM medium with Cy3-BORIS MB (200 nM) and Hoechst 33342 (5 μg/mL) in presence of a Lipofectamine RNAiMAX siRNA transfection reagent (Invitrogen). The cells were washed, resuspended in PBS—5 mM EDTA and cytocentrifugated onto glass slide using a cytospin centrifuge and then examined under a fluorescent (Axioplan2 Imaging, Zeiss) or a confocal (LSM 710 Quasar, Zeiss) microscope.

For ABCG2 fluorescence staining, cells were prepared as described beyond. After cytospin, cells were fixed with ice-cold acetone for 8 min and stained at 4°C overnight with rabbit anti-human ABCG2 antibody (Sigma) used at 1:20 dilution in PBS. Slides were washed with PBS and incubated for 1 hour at room temperature with donkey anti-rabbit secondary antibody labeled with Alexa Fluor 488 (Sigma) used at 1:500 dilution in PBS. The slides were then examined under fluorescent microscope.

### FACS analysis and sorting of SP

HeLa cells (1 x 10^6^ cells/ml) were incubated in serum-free medium at 37°C for 1.5 hour with Hoechst 33342 (Invitrogen) at a final concentration of 12.5 μg/ml either alone or in combination with 50 μM verapamil (Sigma) as a control. The cell suspensions were periodically mixed during the incubation. After incubation, cells were washed with PBS and resuspended in PBS—5 mM EDTA. Before the analysis, the cells were incubated with propidium iodide (2 μg/ml) and filtrated using a cell-strainer of 40 μm mesh size (Falcon). SP analyses were performed using LSRII (Becton Dickinson) and the sorting of SP and NSP (non-SP) using FACS Aria (Becton Dickinson) at the facility of EPFL (Ecole Polytechnique Fédérale of Lausanne). Hoechst 33342 dye was excited at 355 nm and its fluorescence was analyzed using dual-wavelength of emission, 445 nm for Hoechst blue and 650 nm for Hoechst red.

### Ectopic BORIS expression

Hela cells were transfected with pCMV-BORIS plasmid using Lipofectamine 2000 transfection reagent (Invitrogen) as previously described [25].

### BORIS knockdown by inducible shRNA lentiviral system

Stables cell lines with inducible expressing shRNAs targeting human *BORIS* mRNA were prepared as previously described [25]. HeLa, HT29, MCF7 and MDA-MB-231 tumor cell lines were used in these experiments.

### BORIS cDNA expression by inducible lentiviral system

Stable cell lines with inducible expressing human *BORIS* cDNA were generated using the doxycycline-inducible lentiviral system [26]. BORIS cDNA from pCMV-BORIS plasmid was cloned into pINDUCER20 by Gateway Cloning system (Invitrogen). The lentiviral vector pINDUCER20 contains the antibiotic selection marker of G418 (Geneticin), which enables to select only the transduced cells. This vector also contains a cassette with a doxycycline-inducible promoter that controls the transcription of the cloned cDNA. For the generation of lentivirus, we followed our procedure as previously described [25]. The viral suspension combined with 8 μg/ml polybrene (Sigma) was used to infect target cells (HeLa, HT29, MCF7 and MDA-MB-231). Twenty-four hours post infection the medium was replaced with medium supplemented with 500 μg/ml G418 (Roche). After 2 weeks of antibiotic selection, 2 μg/ml of doxycycline (Sigma) was added to the medium to allow induction of *BORIS* cDNA expression and doxycycline-containing medium was refreshed every 3 days.

### Quantitative RT-PCR analysis

qRT-PCR analyses were performed as previously described [25]. The ΔΔCt method was used to determine the relative expression levels, which were normalized to *GAPDH* level. As already reported [25], for all the amplified target genes PCR efficiency extended from 94 to 101%, with correlation coefficient (r2) ranging from 0.96 to 1.0. The Ct values of GAPDH measured the similar levels (Ct = 10.07 ± 0.25) for all the analyzed cells.

The sequences of primer used in addition are shown in S1 Table.

### CD44+/CD24- analysis by flow cytometry

CD44+/CD24- expression was analyzed in cells engineered to stably exhibit knockdown *BORIS* mRNA or express *BORIS* cDNA. Cells were trypsinized and 10^6^ cells were resuspended in 100 μL PBS-1% FBS. Monoclonal mouse anti-human CD44–APC-H7 antibody (BD Pharmingen) and a monoclonal mouse anti-human CD24–Alexa Fluor 647 antibody (BD Pharmingen) were added at dilutions of 1:20 and 1:5 respectively, as suggested by the manufacturer, and incubated for 40 min at 4°C. DAPI was added at concentration of 1 μg/ml during the last 10 min of incubation. After washing with PBS-1% FBS, flow cytometry analysis were performed using Gallios flow cytometer (Beckman Coulter). At least 5 x 10^4^ events were counted for all samples. The analysis of percentage of CD44+/CD24- cells was estimated after excluding dead cells (DAPI positive cells) and gating on eGFP and tRFP positive cells. The results were analyzed using FlowJo software. Three independent experiments were performed.

### Colony forming assay

Cells were trypsinized and resuspended in medium. Three hundred cells were seeded in 6 well/plates in triplicates. Cells were cultured in freshly doxycycline-containing medium. After 2 weeks, cells were fixed with 1 ml of 4% formaldehyde for 10 min at room temperature and stained with 1 ml of 0.1% crystal violet for 10 min. After washing with PBS, each well was photographed and the colonies (determined as >50 cells/colony) were counted using ImageJ program.

### Migration assay

Cell migration was determined using cell culture inserts (BD Falcon) with 8 μm pore size. Briefly, the cells were harvested and resuspended in serum-free medium, 5 x 10^4^ cells were plated into the top of inserts placed in 24-well plates. At the bottom well of the inserts were added 500 μL medium supplemented with 10% FBS. After 48 hours of incubation, the non-migrating cells were removed with a cotton swab and the migrating cells (on the lower surface of the insert) were fixed with 1 ml of 4% formaldehyde and then stained with 1 ml of 0.1% crystal violet for 10 min. After washed 3 times with PBS, the migrating cells were counted under ten random high-power microscopic fields per insert and the mean number of migrating cells was calculated for each group of cells.

### Cell proliferation and chemo-sensitivity assays

Cell proliferation analysis after BORIS silencing and BORIS induction were assessed by MTT assay as previously described [25].

*In vitro* growth effect of 5-Fluorouracil (5-FU) was determined by MTT assay. Briefly, each group of cells, after BORIS silencing, was seeded in triplicate at the density of 1 x 10^4^ cells/well in 96-well plates in doxycycline-containing medium. The day after, 5-FU (Sigma) was added at different concentrations: 0.5, 5, 50 and 500 μg/ml. Cells were incubated for 2 days and then cell viability was measured by MTT assay. Growth inhibition or surviving fraction was expressed as a percentage of the untreated controls that were measured at once, using the equation: (absorbance of treated sample/absorbance of untreated sample) x 100.

### Statistical analysis

Statistical significance was evaluated using two-tailed student t-test analysis. P-value <0.05 was considered statistically significant.

## Results

### *BORIS* mRNA is expressed in side population cells

**Fig 1:**
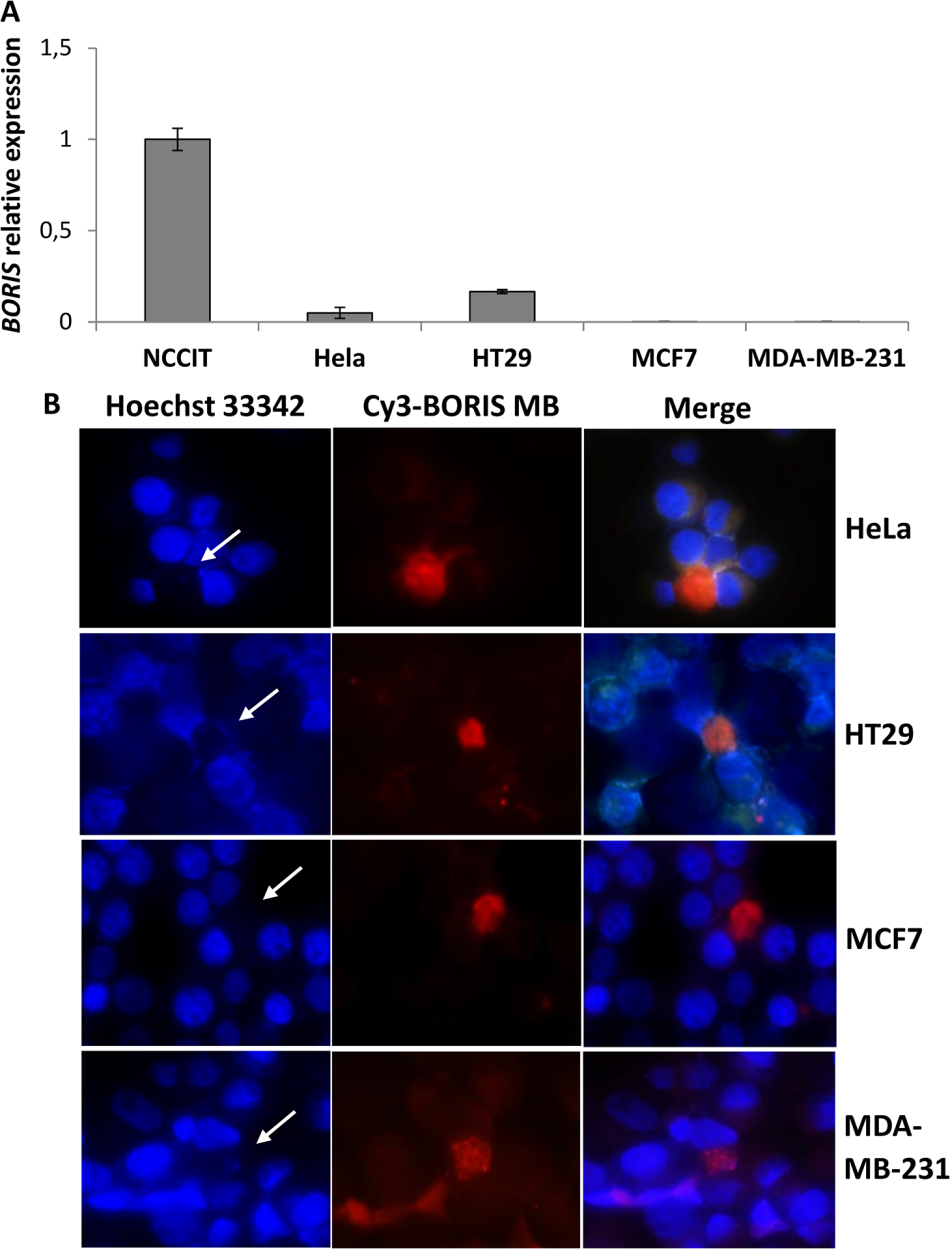
*BORIS* expression and SP analysis in human epithelial tumor cell lines. (A) *BORIS* expression in human tumor cell lines. Total RNA from NCCIT (embryonic), HeLa (cervical), HT29 (colon), MCF7 (non-invasive breast) and MDA-MB-231 (invasive breast) tumor cells were extracted and *BORIS* expression was analyzed by qRT-PCR. The results were normalized to *GAPDH* and related to NCCIT cells. Error bars represent the mean ± SD (n = 3). (B) *BORIS* expression as detected using BORIS-MB. Representative images of HeLa, HT29, MCF7 and MB-MDA 231 cells, 40X magnification. Cells were incubated with Cy3-BORIS MB (200 nM) and Hoechst 33342 (5 μg/mL) at 37°C for 1.5 hour in serum-free medium and then examined under fluorescent microscopy. White arrows indicate Hoechst negative cells which are also *BORIS* mRNA positive as detected by BORIS-MB.

To investigate the presence of *BORIS* mRNA in CSC-enriched populations of epithelial tumor cells, we used as models the human HeLa (cervical), HT29 (colon), MCF7 (non-invasive breast) and MDA-MB-231 (invasive breast) tumor cell lines. Expression analysis showed a lower level of *BORIS* mRNA in HeLa and HT29 compared to embryonic tumor cells (NCCIT), while in MCF7 and MDA-MBA-231 cells *BORIS* mRNA was almost undetectable (Fig 1A). Therefore, HeLa, HT29, MCF7 and MDA-MBA-231 tumor cells can be classified as BORIS-low expressing tumor cells.

Hoechst side population (SP) analysis is a proven technique to enrich stem and early progenitor cells in different cell lines [13–15]. Fluorescence imaging analysis was performed using Hoechst 33342 in combination with *BORIS* mRNA detection using BORIS-Molecular Beacon (BORIS-MB), as previously described [25]. Fluorescence imaging analyses showed that the BORIS positive cells are only a subset of cells in all analyzed epithelial tumor cell lines (Fig 1B), consistent with results obtained in embryonic tumor cells [25]. The estimated frequency of BORIS positive cells is approximately 0.1% in HeLa, 0.5% in HT29 and less than 0.05% in MCF7 and MDA-MBA-231 cells; in contrast it was about 5% in NCCIT [25]. Interestingly, fluorescence imaging analyses showed that the majority of BORIS positive cells were also Hoechst negative (white arrows in Fig 1B and S1 Fig). Therefore, *BORIS* expression could be associated with Hoechst negative phenotype of SP cells in cervical, colon and breast tumor cells. However, a small percentage (less than 10%) of BORIS positive cells were Hoechst positive.

**Fig 2:**
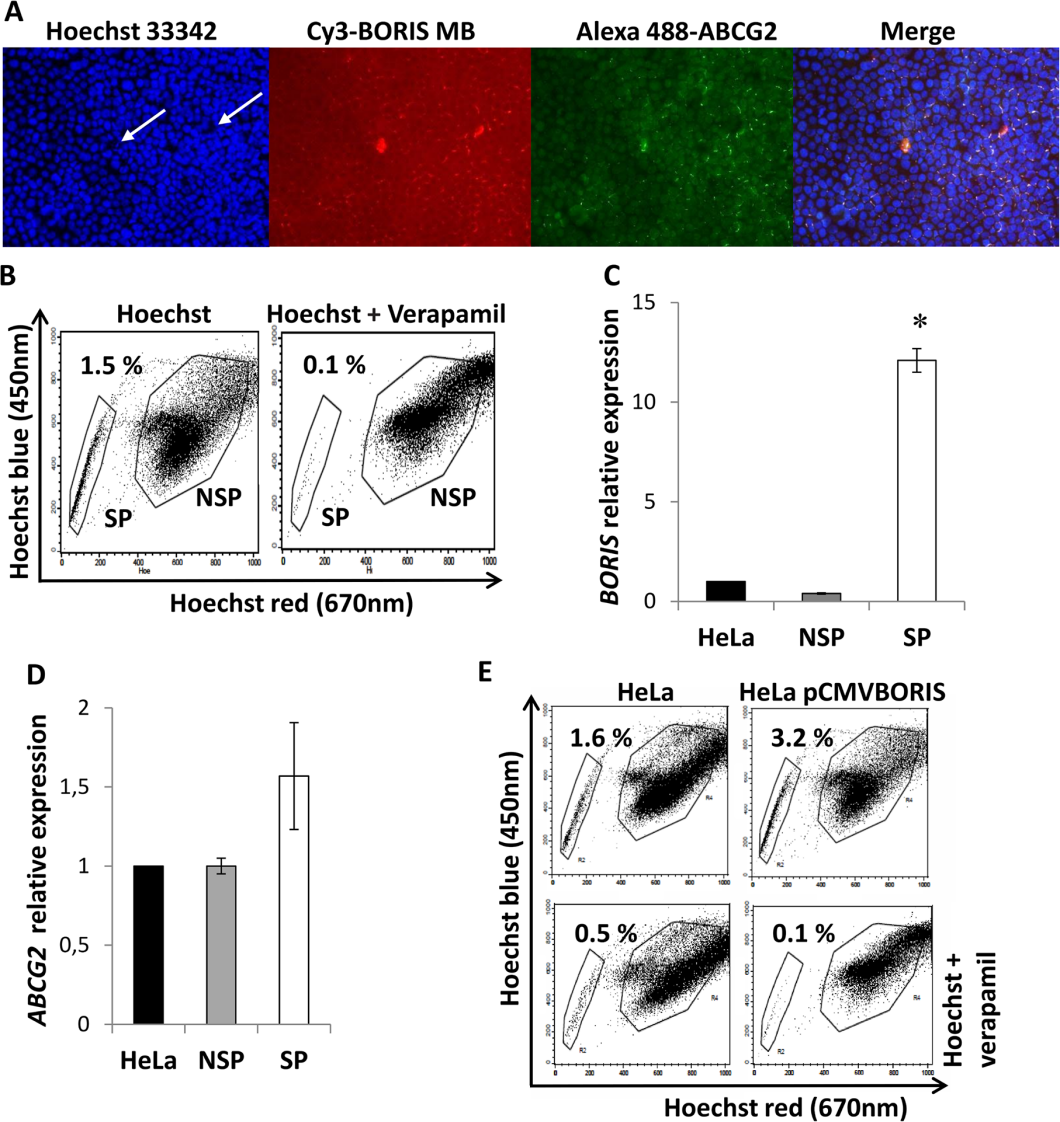
*BORIS* and *ABCG2* expression in isolated SP HeLa cells. (A) Immunofluorescence analysis of ABCG2 in HeLa cells. The cells were incubated with BORIS-MB and Hoechst 33342 at 37°C for 1.5 h in serum-free medium. After cytocentrifugation the slides were fixed with cold acetone and then incubated with rabbit polyclonal ABCG2 antibody. The BORIS positive/ABCG2 positive/Hoechst negative cells are indicated with white arrows. 10X magnification. (B) Representative dot plot of flow cytometry analysis of SP. HeLa cells were incubated with Hoechst 33342 (12.5 μg/mL) either alone or in combination with verapamil (50 μM). The analysis was performed using LSRII and the sorting using FACS Aria. The gates indicate the sorted SP and NSP cells. (C) *BORIS* mRNA and (D) *ABCG2* mRNA expression in SP and NSP isolated from HeLa cells. Total RNA was extracted and analyzed by qRT-PCR. Graphs indicate the mRNA levels of *BORIS* and *ABCG2* genes normalized to *GAPDH* and related to the parental HeLa cells. Data are represented as mean ± SD from 3 independent experiments. Asterisk indicates p<0.05. (E) SP analysis in BORIS overexpressed cells. HeLa cells were transiently transfected with a BORIS expression vector (HeLa pCMVBORIS). After 2 days, 1 x 10^6^ cells were incubated with Hoechst 33342 (12.5 μg/mL) either alone (top) or in combination (bottom) with 50 μM verapamil. The analysis was performed using LSRII flow cytometry and one representative experiment of 3 independent experiments is shown.

ABCG2 is described as the major efflux pump responsible for Hoechst negative phenotype in SP cells [27]. Hence, a possible co-expression of *BORIS* with the chemoresistance transporter protein ABCG2, was first investigated. HeLa cells were used in this experiment. As can be seen in Fig 2A, BORIS positive cells are both negative for Hoechst (white arrows) and positive for ABCG2 protein. To confirm the presence of *BORIS* mRNA in CSC-enriched population of HeLa cells, the SP cells and also the non-SP (NSP) cells were sorted by FACS. The percentage of SP cells was from 0.5% to 1.5% of the total cells (Fig 2B), consistent with previous reports [15, 27]. The SP fraction was completely reduced by adding verapamil, an inhibitor of the ABC-transporters, indicating that the population was bona fide SP cells. The qRT-PCR analysis showed that *BORIS* mRNA level of SP cells was significantly higher (12 fold, p<0.05) compared to NSP and parental HeLa cells (Fig 2C). Also the *ABCG2* mRNA level was higher (about 1.5 fold) in SP sorted cells compared to NSP and parental cells (Fig 2D). To further verify the presence of *BORIS* in the SP CSC-enriched population, the frequency of SP was analyzed in pCMVBORIS-transfected HeLa cells (Fig 2E). As expected, the overexpressing BORIS cells were significantly more enriched in SP cells (2 fold, p = 0.01) compared to HeLa parental cells. All these results indicated that the isolation of BORIS-positive cells could lead to an enrichment of the CSC population in HeLa tumor cells.

### Colon-spheres and mammo-spheres express high levels of *BORIS* mRNA

**Fig 3:**
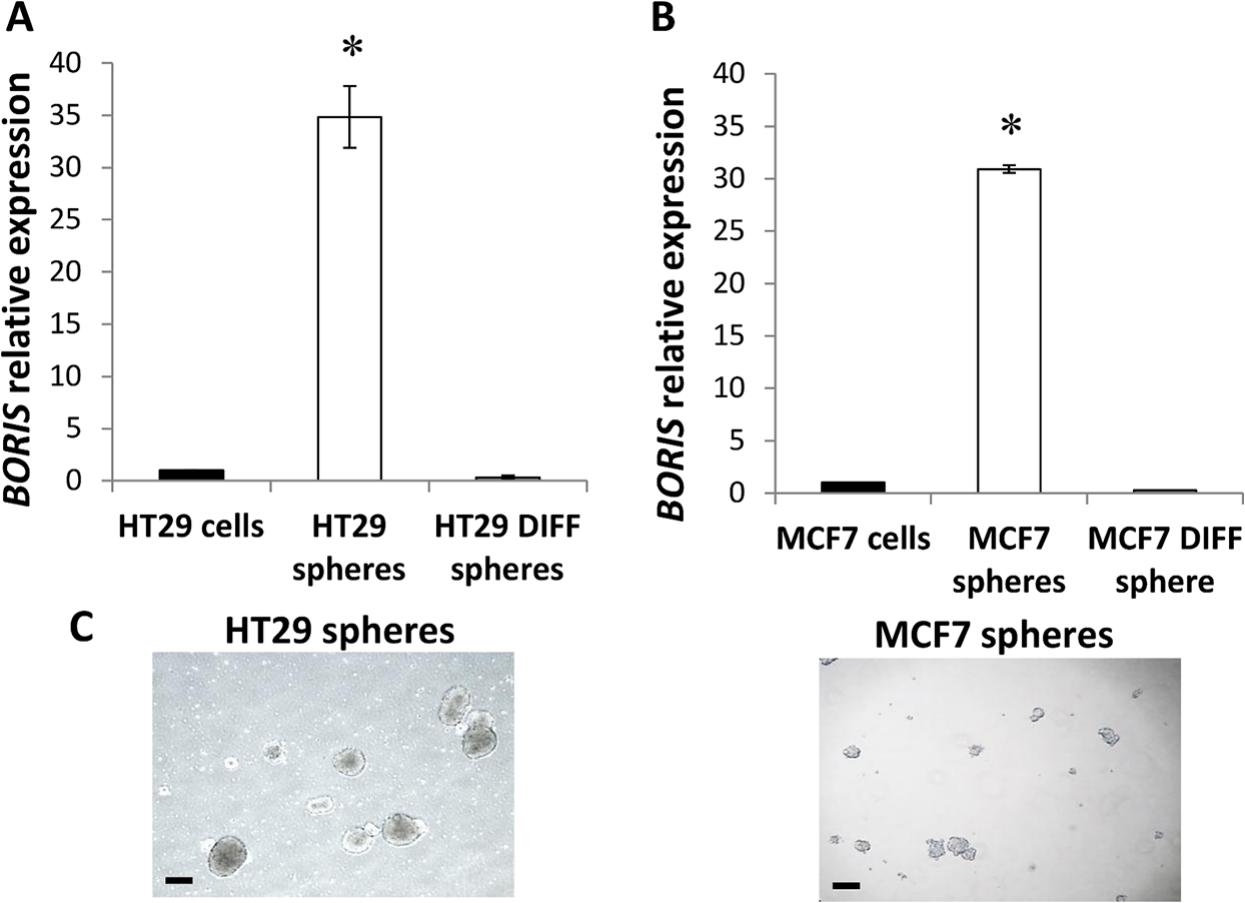
*BORIS* expression in colon-spheres and mammo-spheres. Cells were seeded at low density (1,000 cells/ml) in sphere culture medium in low attachment plates. After 10–15 days spheres were collected for RNA extraction and an aliquot of spheres was seeded in normal medium with serum to allow the differentiation (differentiated-spheres). *BORIS* expression was analyzed by qRT PCR in (A) colon-spheres (HT29 spheres) and differentiated-spheres (HT29 DIFF spheres) and in (B) mammo-spheres (MCF7 spheres) and differentiated-spheres (MCF7 DIFF spheres). Data were normalized to *GAPDH* and related to parental cells (cells). One representative experiment of 4 independent experiments is shown. Asterisks indicate statistically significant difference (p<0.05) between spheres and parental cells. (C) Representative images of colon-spheres and mammo-spheres are shown, 4X magnification. Black scale bars indicate 250 μm.

The growth of cells in suspension, with a serum-free medium (sphere culture), is a common approach to CSC-enrichment [7–8]. As this property was reported to be restricted to stem/progenitor cells [19], *BORIS* expression was also investigated in forming-spheres of colon (HT29) and breast (MCF7) tumor cells. An aliquot of spheres were seeded in serum-containing medium to allow the differentiation. Interestingly, *BORIS* expression analysis revealed a significant higher level of *BORIS* mRNA in colon-spheres (37.2 ± 7.3 fold, n = 4) as well as in mammo-spheres (30.9 ± 6.4 fold, n = 4), compared to parental cells and differentiated-spheres (Fig 3). These results suggested that *BORIS* is expressed in the CSC-enriched spheres of colon and breast tumor cells.

### Gene expression and CSC phenotype in BORIS knockdown cells

To explore a possible role of BORIS in CSCs, we selected a knockdown strategy using lentiviral system with inducible expressing shRNAs targeting human *BORIS* mRNA. The BORIS sh-3, BORIS sh-4 and scrambled-shRNA (CTR sh) lentiviruses were previously generated and validated [25]. These lentiviruses were used to infect HeLa, HT29, MCF7 and MBA-MD-231 tumor cells.

**Fig 4:**
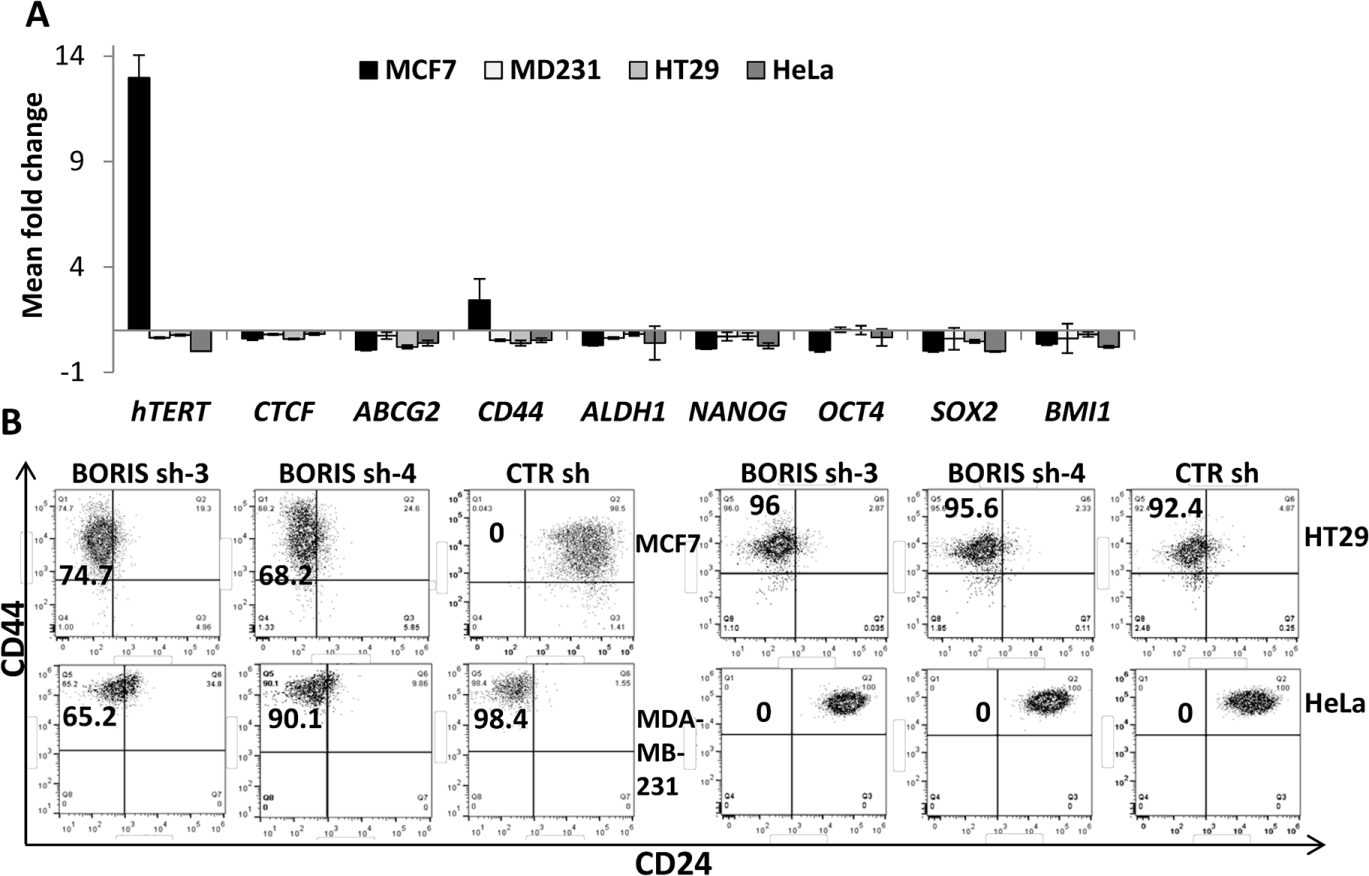
Impact of BORIS-knockdown on gene expression and CSC profile. (A) MCF7, MDA-MB-231, HT29 and HeLa cells were engineered to stably exhibit knocked-down *BORIS* mRNA. BORIS sh-3, sh-4 and CTR sh (control shRNA containing scrambled sequence) lentiviruses were used to infect these cells. Each transduced cells were cultured with doxycycline to induce BORIS shRNA expression. Medium containing doxycycline was replaced every 3 days. After 2 weeks, RNA was isolated from BORIS sh-3, sh-4 and CTR sh of each transduced cell line. mRNA levels of the indicated genes were analyzed by qRT-PCR. Graphs represent for each gene the means of fold induction of both BORIS shRNA (BORIS sh-3 and sh-4) related to that of control of any cells. Standard errors were calculated considering error propagation of both BORIS shRNA analyses. Graphs show one representative experiment. (B) Representative flow cytometry dot plots of CD44 and CD24 expression in MCF7, MDA-MB-231, HT29 and HeLa cells engineered to stably exhibit knocked-down *BORIS* mRNA. CD44 and CD24 expression patterns of the two BORIS shRNA (BORIS sh-3 and sh-4) and the control (CTR sh) are shown. Anti-CD24 antibody labeled with AlexaFluor 647 and anti-CD44 antibody labeled with APC-H7 were used. The percentage of CD44+/CD24- population was estimated after gating on eGFP and tRFP positive cells (transduced and dox-induced shRNA, respectively) and the final gates are based on the isotype control corresponding to each cell line. All experiments were conducted independently three times and one representative experiment is shown for each group of cells.

It has been demonstrated that BORIS activates *hTERT* expression [28] and affects the expression of stemness genes in embryonic tumor cells [25]. In this study, these correlations were explored in epithelial tumor cells. After 2 weeks of BORIS knockdown, expression analyses of *hTERT*, *CTCF*, the most common CSC markers (*ABCG2*, *CD44*, *ALDH1*) and stem cell (*NANOG*, *OCT4*, *SOX2*, *BMI1*) genes were performed. Fig 4A shows, for all analyzed genes, the mean of the fold change of BORIS sh-3 and BORIS sh-4 cells compared to CTR sh cells of each engineered tumor cell line. *hTERT* expression was significantly down-regulated in all analyzed tumor cells at the exception of MCF7, in which a significant up-regulation (13 fold) was observed. *CTCF* expression was decreased in all cells, even if the decrease was not significant (from 40% to 20% compared to control). Notably, absence of BORIS trigged a decrease of the *ABCG2* expression (93% for MCF7, 25% for MDA-MB-231, 80% for HT29 and 60% for HeLa). *CD44* was down-regulated in all but one cell line, MCF7, in which a 2.5 fold increase was observed. *ALDH1*, *NANOG*, *OCT4*, *SOX2* and *BMI1* were generally down-regulated in all tumor cells after BORIS silencing. All together, these results suggested that BORIS could significantly affect the regulation of *hTERT*/telomerase, stem cell and CSC marker genes in epithelial tumor cells.

CD44+/CD24- subpopulation has been found to be enriched with tumor-initiating features, especially in breast cancer cells [6, 29]. In this study, CSC subpopulation was analyzed by flow cytometry in BORIS-knockdown tumor cells. A different behavior of MCF7 compared to the other cells was observed (Fig 4B). The analyses revealed a remarkable acquisition of CD44+/CD24- phenotype in BORIS-knockdown MCF7-derived cells, from none CD44+/CD24- cells in the control to about 70% in the BORIS-knockdown cells. A non significant decrease of CD44+/CD24- subpopulation was observed in BORIS-shRNA MDA-MB-231-derived cells. In the non-breast tumor cells, HT29 and HeLa, no change of expression was observed with both cells displaying a typical CD44+/CD24- epithelial phenotype.

### Knockdown of BORIS affects cell proliferation in MCF7 breast cells

**Fig 5:**
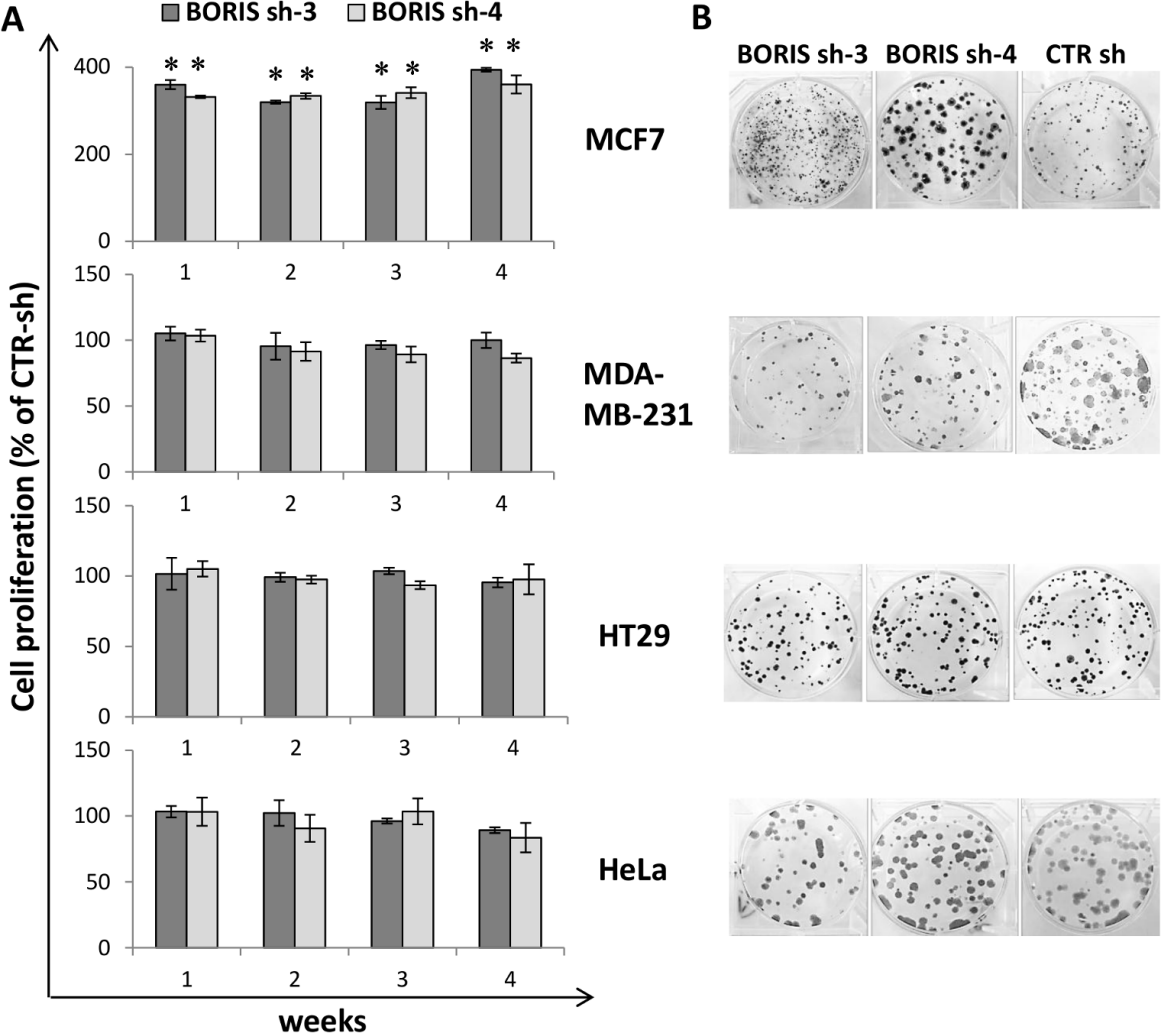
Impact of BORIS knockdown on cell survival in epithelial tumor cells. (A) Cell proliferation, over 1 month of dox-induced BORIS- and CTR- shRNA cells, was analyzed each week by MTT assay. Results of the two specific BORIS-shRNA (BORIS sh-3 and sh-4) are indicated as a percentage compared to the cell proliferation of control cells (scrambled shRNA, CTR sh). Error bars represent the mean ± SD (n = 3). Asterisks indicate statistically significant difference (p<0.05) between BORIS sh and CTR sh. (B) Representative images of colony formation assay after 1 month of BORIS knockdown. Three hundred cells were seeded in each well of 6-well plates with medium containing doxycycline, each cell group were prepared in triplicate. Cells were cultured for 2 weeks, then were fixed and stained with crystal violet.

Cell survival was assessed following BORIS knockdown. Cell proliferation and the capacity to form colonies were measured each week for one month. The colony formation (or clonogenic) assay monitors the ability of a cancer cells to produce a viable colony after treatment [30] and was used to analyze the *in vitro* capacity of tumor cells to form colonies after BORIS knockdown. Results showed no significant difference on cell proliferation in all but one tumor cell line. The exception concerned MCF7 cells in which a significant increase (3–4 fold, p<0.0001) of cell proliferation compared to control cells was observed (Fig 5A). Colony formation assays confirmed the cell proliferation results (Fig 5B). After one month of BORIS-knockdown, the numbers of colonies were not significantly different for HeLa, HT29 and MDA-MB-231, compared to controls. In contrast, the number of colonies was significantly higher in MCF7. These results indicated that, at the exception of MCF7 breast cancer cells, BORIS silencing in epithelial tumor cells did not have a significant impact on cell survival.

### Knockdown of BORIS impairs the sphere formation capacity of colon and breast tumor cells

**Fig 6:**
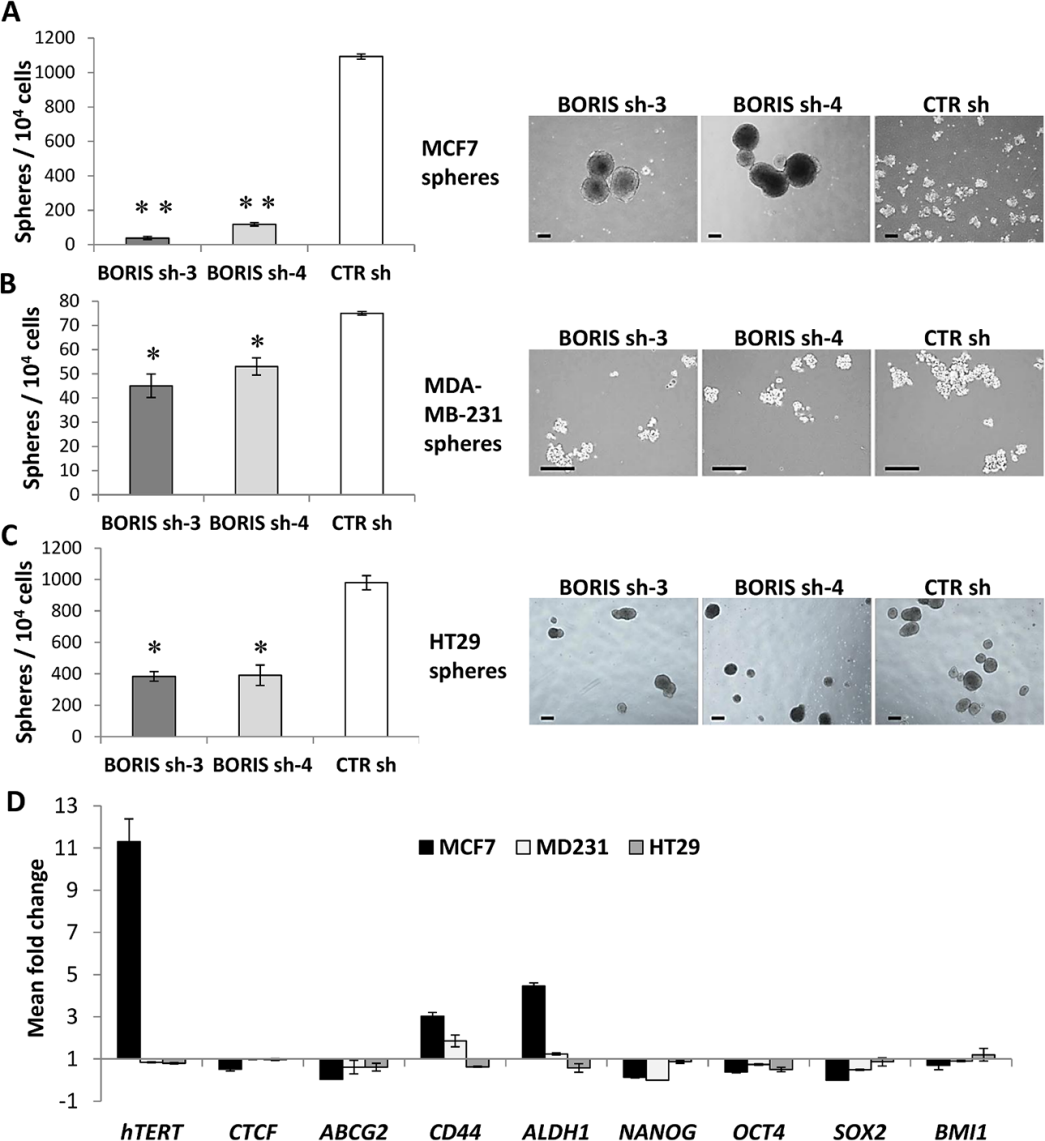
Knockdown of BORIS reduces the ability to form spheres and down-regulates the expression of stem cell genes. (A) MCF7, (B) MDA-MB-231 and (C) HT29 cells were engineered to stably exhibit knocked-down *BORIS* mRNA. After 2 weeks of dox-induction of BORIS- and CTR- shRNA cells were seeded at low density (1000 cells/ml) in sphere serum-free medium into ultra-low attachment 6-well plates in triplicates. Doxycycline was added every 3 days to maintain shRNA induction. Formed spheres were counted after 10 days. Error bars represent the mean ± SD (n = 3). One asterisk (p<0.05) or two asterisks (p<0.001) indicate statistically significant difference between BORIS sh and CTR sh spheres. On the right are shown representative images of spheres. MCF7-spheres and HT29-spheres, 4X magnification. MDA-MB-231-spheres, 10X magnification. Black scale bars indicate 250 μm. (D) Expression analysis of BORIS-depleted spheres. Total RNA was isolated from BORIS sh-3, sh-4 and CTR sh formed spheres. mRNA levels of the indicated genes were analyzed by qRT-PCR. Graphs represent for each gene the means of fold induction of both BORIS shRNA (BORIS sh-3 and sh-4) related to that of control. Standard errors were calculated considering error propagation of both BORIS shRNA analyses. Graphs show one representative experiment.

The impact of BORIS knockdown on self-renewal capacity of tumor spheres was also considered. Notably, the total number of tumor spheres was significantly decreased in all BORIS-shRNA derived cells (Fig 6). The number of spheres formed by BORIS-shRNA engineered derived cells was significantly decreased compared to control (90–95% for MCF7, 30–40% for MDA-MB-231 and 60% for HT29). Representative images of spheres show that the size was similar between BORIS-sh and CTR sh derived spheres for MDA-MB-231 and HT29 (Fig 6B and 6C, right). However, the size of spheres of BORIS-shRNA MCF7-derived cells were larger compared to control cells (Fig 6A, right), the sphere diameters were ≥ 400 μm and ≤ 200 μm, respectively. Interestingly, it emerged that BORIS depletion had a negative effect on tumor sphere formation capacity in colon and in breast cancer cells in which the number of spheres were dramatically reduced. This result indicated that BORIS could play a role in the self-renewal capacity of colon and breast cancer cells.

Gene expression profile analysis was also investigated in formed spheres after BORIS silencing. The spheres formed by HT29 and MDA-MB-231 BORIS-depleted cells showed a down-regulation (15–20%) of *hTERT* expression compared to controls (Fig 6D). In contrast, BORIS depleted-MCF7 spheres displayed a strongly increase of *hTERT* expression (11 fold). No significant difference was observed for *CTCF* in the different tumor cell lines. These observations were consistent with the findings already observed in BORIS knockdown cells (Fig 4A). The expression profile of stem cell and CSC markers genes of BORIS-depleted spheres showed that *NANOG*, *OCT4* and *SOX2* genes were down-regulated in both breast and colon tumor cells. *ABCG2* expression was decreased for all BORIS-silenced spheres. *CD44* and *ALDH1* were up-regulated in BORIS-depleted spheres of breast tumor cells (3.7 for MCF7, 1.5 fold for MDA-MB-231) but down-regulated in HT29 BORIS-depleted spheres. All these results demonstrated that the phenotype could be dependent of the origin of the tumor cells.

### Knockdown of BORIS up-regulates epithelial-mesenchymal-transition (EMT)-related genes in MCF7 cells

**Fig 7:**
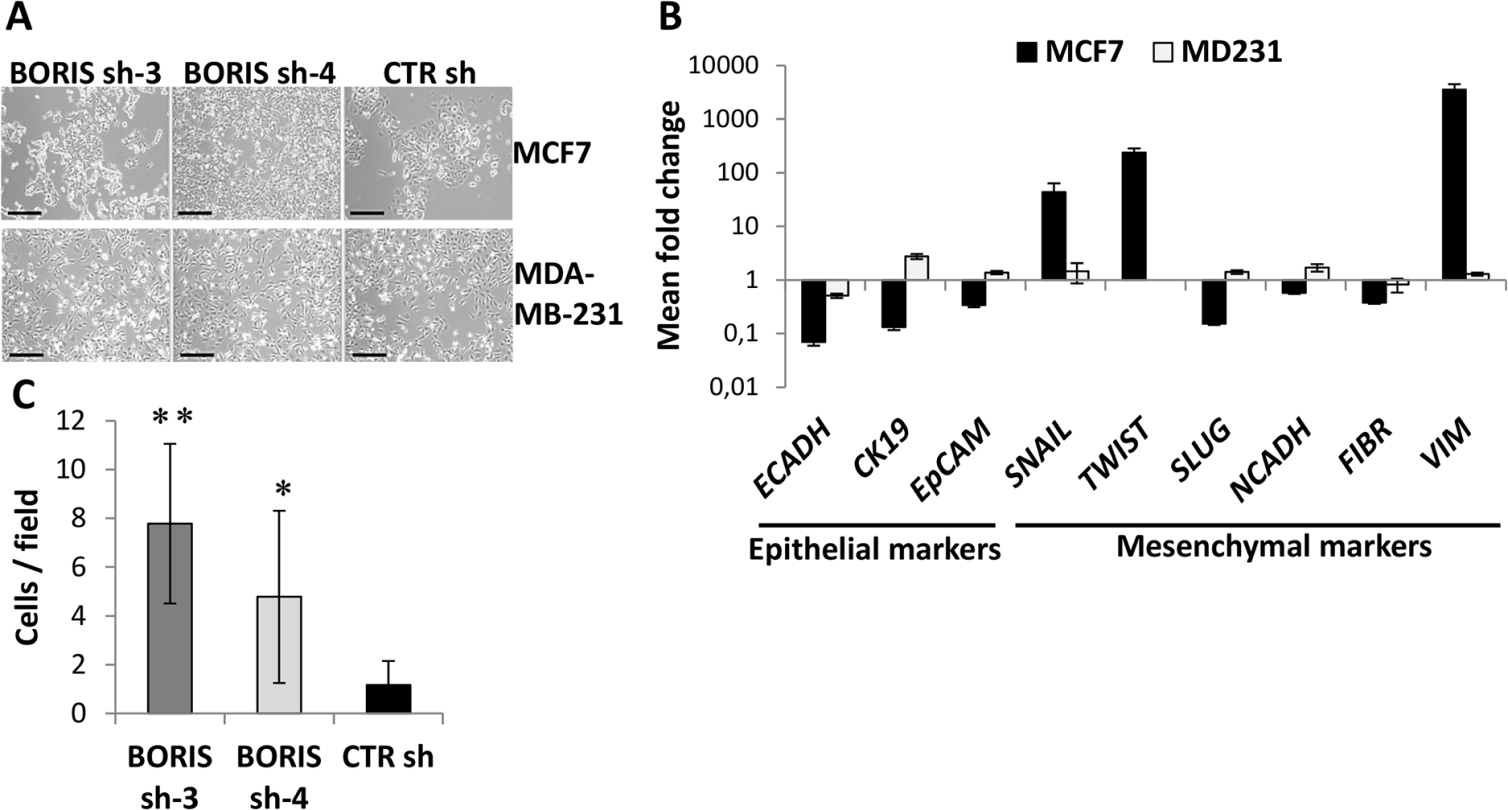
Acquisition of EMT phenotype and gene signature of MCF7 cells after BORIS silencing. (A) Representative images of MCF7- and MDA-MB-231- derived cells after dox-induction of BORIS- and CTR- shRNA, 10X magnification. Black scale bars indicate 250 μm. (B) After 2 weeks of BORIS knockdown, mRNA levels of the indicated genes were analyzed by qRT-PCR. Graphs represent for each gene the means of fold induction of both BORIS shRNA (BORIS sh-3 and sh-4) related to the control. Standard errors were calculated considering error propagation of both BORIS shRNA. Graphs show one representative experiment. Results are shown in logarithmic scale. (C) Cell migration assay of MCF7 cells after BORIS knockdown. Graph shows the mean of cell number visualized in 10 different fields ± SD (n = 3). One asterisk (p<0.05) or two asterisks (p<0.001) indicate statistically significant difference between BORIS sh and CTR sh. On the right, representative images, 10X magnification.

In BORIS-depleted MCF7 cells, we observed an increase of *hTERT* transcription, an acquisition of the CSC phenotype (CD44+/CD24-) and an increase of cell survival. Interestingly, a change in morphology was also noticed (Fig 7A). These cells formed structures with irregular shape and unique spindle morphology. This particular morphology is characteristic of mesenchymal cells, such as MDA-MB-231 cells (Fig 7A), thus we further investigated whether an alteration of the genes controlling the epithelial–mesenchymal transition (EMT) cellular process could occur in MCF7 cells. Expression of a panel of representative genes, which act during the EMT program, were analyzed by qRT-PCR after BORIS silencing (Fig 7B). Interestingly, it emerged that BORIS-depleted MCF7 cells acquired EMT gene signature. As expected, the mesenchymal MDA-MB-231 cells neither changed of morphology (Fig 7A) nor modified significantly EMT gene expression profile (Fig 7B). In contrast, MCF7 cells after BORIS silencing lost the expression of epithelial markers, such as cytokeratin-19 (CK19), epithelial cellular adhesion molecule (EpCAM) and especially E-cadherin (ECADH). Simultaneously, we observed a remarkable up-regulation of several mesenchymal markers, including SNAI1 (SNAIL), Twist-related protein 1 (TWIST) and vimentin (43, 238 and 3567 fold increase, respectively). No significant change in expression was noticed in the other EMT-related genes, SNAI2 (SLUG), N-cadherin and fibronectin. All these results suggested that BORIS could affect the EMT process in luminal-like non-invasive breast cancer cells and indicated that BORIS may regulate some of the EMT-related genes, such as *CDH1 (ECADH)*, *SNAIL*, *TWIST* and *VIMENTIN*. To confirm the acquisition of EMT phenotype of MCF7 cells after BORIS knockdown, we analyzed the migration capacity of these cells. As expected, the non-invasive breast MCF7 cells, which normally do not migrate, acquired the capacity to migrate after BORIS silencing (Fig 7C). This last finding confirmed the acquisition of EMT gene signature of MCF7 cells after BORIS silencing.

### Cell viability in BORIS knockdown-derived cells after 5-FU treatment

The chemoresistance is a characteristic feature of CSCs [3] and it was reported that an increase of hTERT, EMT genes and CD44+/CD24- expression allow to an enhancement of resistance to chemotherapeutic drugs [31–33]. In BORIS inhibition experiments, an increase of hTERT, EMT genes expression and an acquisition of CD44+/CD24- phenotype were seen in MCF7 cells. To better analyze the effects of BORIS silencing in the analyzed epithelial tumor cells, the impact of treatment with a chemotherapeutic drug was also analyzed. The 5-Fluorouracil (5-FU) is one of the most used drugs in chemotherapy. This drug is used to treat several cancers including colon, breast, ovarian, head and neck and liver cancer [34].

**Fig 8:**
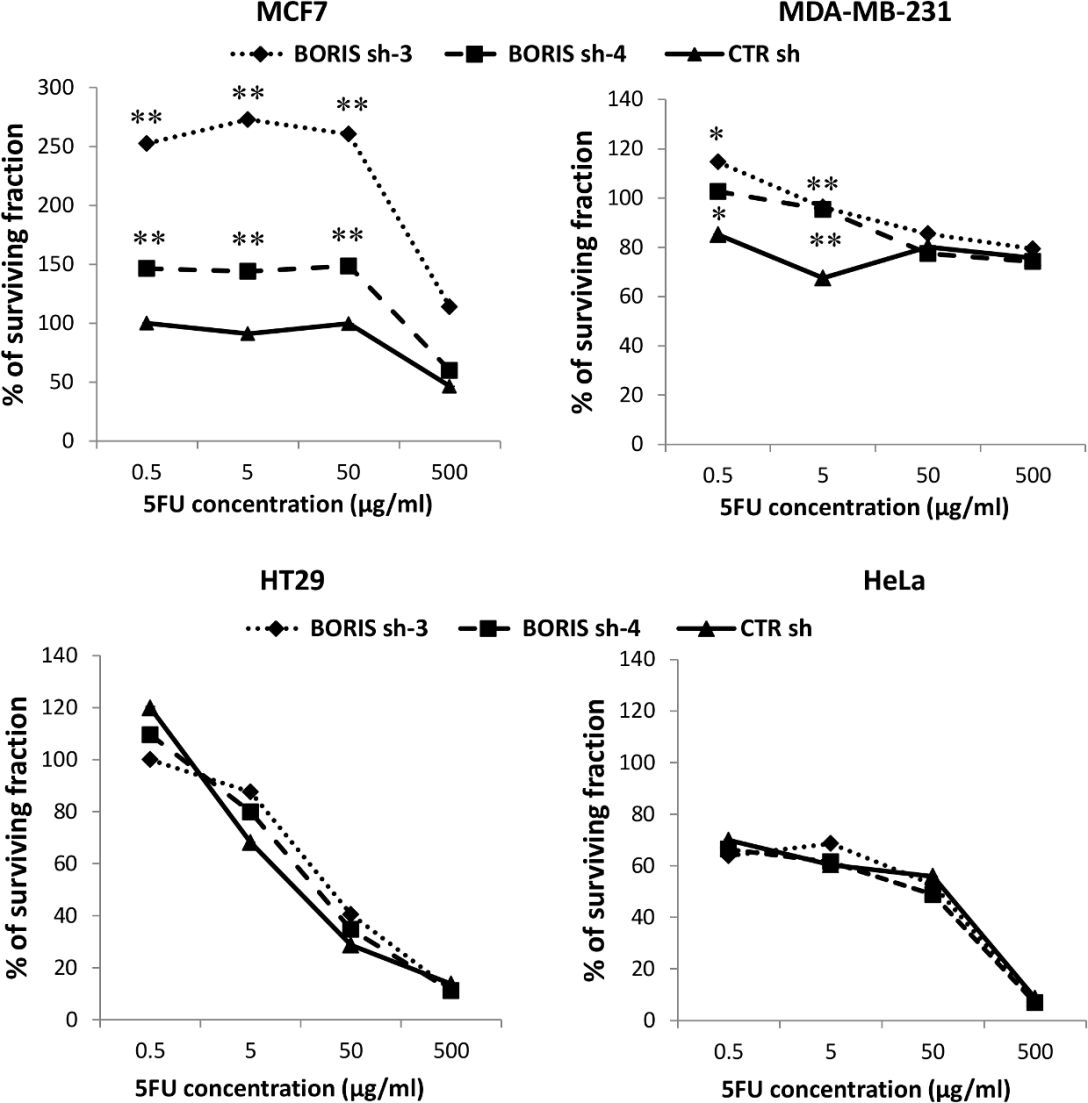
Effect of 5-FU on cell proliferation of BORIS-depleted tumor cell lines. After 2 weeks of BORIS knockdown cells were seeded (1 x 10^4^ cells/well) in 96-well plates in doxycycline-containing medium. The day after was added 5-FU at different concentrations: 0.5, 5, 50 and 500 μg/ml. Cells were incubated for 2 days and then cell viability was measured by MTT assay. The percentage of viable cells (% of surviving fraction) is shown relative to that of the untreated control. Error bars represent the mean ± SD (n = 3). One asterisk (p<0.05) or two asterisks (p<0.01) indicate statistically significant difference between BORIS sh and CTR sh cells.

After 2 weeks of BORIS silencing, the derived tumor cells were treated with different concentration of 5-FU (0.5, 5, 50 and 500 μg/ml). The cell viability was determined by MTT assay and was expressed as the percentage of surviving 5-FU-treated cells compared to non-treated cells (Fig 8). As expected, BORIS-depleted MCF7 cells were significantly (p<0.01) more resistant to 5-FU compared to the control cells. BORIS-depleted MDA-MB-231 cells acquired chemoresistance only at low concentrations (0.5 and 5 μg/ml) of 5-FU. For HT29 and HeLa cells the treatment with 5-FU did not change the cell viability of the BORIS-depleted cells.

### The effects of induction of *BORIS* expression on cell growth and *hTERT* expression

**Fig 9:**
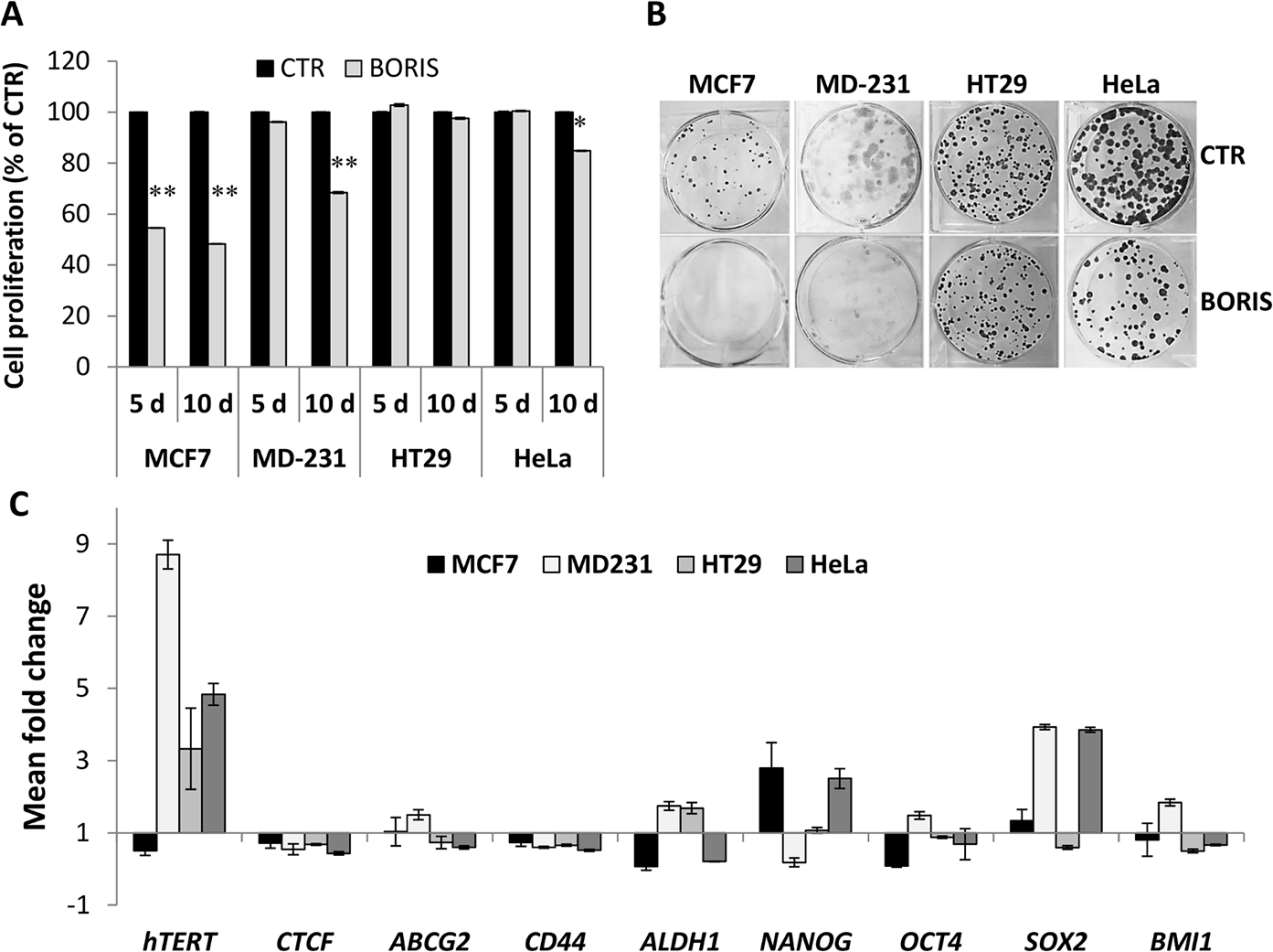
Inhibition of cell growth and up-regulation of *hTERT* and stem cell genes expression, after BORIS-induction in epithelial cancer cells. MCF7, MDA-MB-231, HT29 and HeLa cells were engineered to stably express BORIS cDNA. After transduction with either lentivirus harboring BORIS cDNA (BORIS) or control lentivirus (CTR), cells were selected by incubation with the antibiotic G418 for at least 2 weeks. (A) Cell proliferation was analyzed by MTT assay after 5 and 10 days of dox-induction of BORIS expression. Results are indicated as a percentage compared to the control cells (CTR). Error bars represent the mean ± SD (n = 3). One asterisk (p<0.05) or two asterisks (p<0.01) indicate statistically significant difference between BORIS and CTR cells. (B) Representative images of colony formation assay. Three hundred cells were seeded in each well of 6-well plates with medium containing doxycycline, each assay was performed in triplicate. Cells were cultured for 2 weeks, then were fixed and stained with crystal violet. (C) After 2 weeks of dox-induction of BORIS and CTR cells, mRNA levels of the indicated genes were analyzed by qRT-PCR. Graph represents for each gene the fold induction of BORIS-induced cells related to control cells. Error bars represent the mean ± SD (n = 3).

In order to further investigate the biological effects of BORIS in epithelial tumor cells, we generated stable cells in which *BORIS* cDNA was expressed under the control of doxycycline. Interestingly, at the exception of HT29, the analysis of cell proliferation showed a significant decrease of cell growth after BORIS-induction (Fig 9A). The inhibition of cell proliferation was dramatically affected in MCF7 cells, already after 5 days of BORIS induction. These results were in agreement with the analysis of colony formation (Fig 9B), which demonstrated a significant decrease of clonogenic cell potential. These data suggested a role of BORIS in tumor cell proliferation as a tumor suppressor gene in cervical and breast tumor cells.

Cell morphology was not modified after BORIS induction (data not shown). The analysis of expression profile, 10 days after BORIS induction, showed that *hTERT* expression was significantly increased in MDA-MB-231, HT29 and HeLa cells (8.7, 3.3 and 4.8 fold, respectively) (Fig 9C). In contrast, a decrease of *hTERT* expression was observed in MCF7 cells. These findings are in correlation with the data found in BORIS-depleted cells in which *hTERT* expression was changed in opposite manner. No significant change of expression was found for *CTCF*, *ABCG2* and *CD44*. For *ALDH1* and stem cell genes, a cell-dependent behavior was observed. Indeed, for certain cells, a significant up-regulation was noticed for *SOX2* and *NANOG*. The analysis of CD44+/CD24- expression profile showed that BORIS-induced cells did not have a different phenotype compared to control cells (data not shown).

All these data further confirmed that BORIS could play an important role in the regulation of *hTERT* expression and stem cell genes but in a cell-type dependent manner.

## Discussion

Our data demonstrated that BORIS-positive cells are only a small subpopulation (0.02–0.5%) and this cell subset could be CSC-enriched. Furthermore, high *BORIS* expression was found in the CSC-enriched populations isolated from HeLa SP cells, colon-spheres and mammo-spheres. These observations are consistent with recent reports showing a correlation between *BORIS* expression and advanced stage in different cancers [21–24]. In hepatocellular carcinoma tissues, a correlation between BORIS expression and the CSC marker CD90 has also been observed [24].

The potential role of BORIS in CSCs may arise from the functional studies conducted in this study. Analysis of gene expression profiles after BORIS silencing revealed significant down-regulation of *hTERT*, stem cell and CSC marker genes in the analyzed cells and also in derived spheres. These results, in accordance with our previous study in embryonic cancer cells [25], demonstrate that BORIS can regulate the expression of *hTERT*, stem cell and CSC marker genes not only in embryonic cancer but also in epithelial tumor cells. This role of BORIS could explain the significant reduction of the capacity to form spheres in colon and breast tumor cells, indicating a putative involvement of BORIS in the self-renewal of tumor cells. Nevertheless, BORIS silencing did not lead to a significant inhibition of cell proliferation, even after a relatively long time of BORIS silencing (2 months, data not shown). This result could be explained by the fact that the analyzed tumor cells are BORIS-low expressing cell lines. Hence, an inhibition of BORIS in a small subpopulation of BORIS positive cells would take long time to have a significant impact on cell proliferation of the entire bulk of tumor cells. The discrepancy with the significant reduction of spheres could be due to the different conditions in which the analyses were done. Indeed, the cell viability analyses were measured at steady state and included all the bulk tumor cells, while the sphere cultures create experimental conditions that are favorable to stem cells.

Our results are apparently inconsistent with a previous work which has shown an increase of apoptosis after BORIS-knockdown in MDA-MB-231 cells [35]. This difference could be due to the different experimental conditions and settings. In our study, the experiments have been designed to minimize the risk of false or ambiguous results. For each cell line, we generated two different stable BORIS-depleted cells, and the analyses were performed after 2 weeks of BORIS silencing. Notably, all the assays were executed with FACS-isolated transduced cells. Furthermore, the selected BORIS shRNAs do not overlap with the homolog sequences of CTCF and target the exon 9, which is present in almost all the *BORIS* mRNA isoforms [36].

A completely different phenotype, after BORIS knockdown, was observed in the non-invasive MCF7 breast tumor cells. We observed a remarkable different morphology in both two stable BORIS-shRNA engineered MCF7-derived cells compared to control cells and a significant enhancement of cell proliferation. The gene expression profile analysis revealed a notable up-regulation of *hTERT*, *CD44* and *ALDH1* genes. Importantly, an acquisition of the CSCs phenotype (CD44+/CD24-) and an increase of chemoresistance after 5-FU treatment were observed. Although, a decrease of sphere formation capacity was observed, the size of spheres was larger compared to those of control and this observation can be correlated with the morphologic modification of the MCF7 cells after BORIS silencing. Since a morphologic change to a mesenchymal phenotype was detected in MCF7 cells, we further explored the change of expression of genes involved in the EMT program. A significant up-regulation of the most important mesenchymal marker genes (*SNAIL*, *TWIST* and *VIMENTIN*) and a down-regulation of epithelial genes (*E-cadherin*, *Cytokeratin-19* and *EpCAM*) were observed. The increase of migration capacity confirmed the acquisition of EMT signature. Interestingly, a remarkable increase of *hTERT* expression was also observed in BORIS-depleted MCF7cells. This up-regulation of *hTERT* could be an explanation for the phenotype modification observed in MCF7 cells. Indeed, it has been demonstrated that hTERT/telomerase has also telomere-independent functions [37] and an overexpression of *hTERT* led cells more resistant to several insults, such as treatment with chemotherapeutic [31–32]. An increase of chemoresistance was detected in BORIS-depleted MCF7cells. An enhancement of cell proliferation was also observed, which is in accordance to previous reports in which hTERT was ectopically expressed [28, 38, 39]. Recent studies have shown that hTERT can also act as a transcriptional modulator of the Wnt/β-catenin signaling pathway [40–41] which is able to regulate the EMT program. As well, Wnt signaling aberrantly drives EMT genes to tumor formation in experimental models [42]. Cells that undergo the EMT process acquire the CD44+/CD24- expression pattern and the ability to form spheres [33]. Indeed, an acquisition of CSCs phenotype and the ability to form bigger spheres were found in BORIS-depleted MCF7 derived cells. Further studies analyzing the tumorigenic potential of these derived BORIS-depleted MCF7 spheres may highlight their biological functions. Additionally, we observed a significant down-regulation of *CDH1* (E-cadherin) gene. The adhesive glycoprotein E-cadherin is the master-regulator of the epithelial phenotype and its loss is considered a hallmark of EMT. *CDH1* has been shown transcriptionally silenced by its transcriptional repressors, including SNAI1 (SNAIL), SNAI2 (SLUG) and TWIST [43]. Another important marker of EMT, which was up-regulated in BORIS-depleted MCF7 cells, is *VIMENTIN*, a major constituent of the intermediate filament family of proteins. VIMENTIN is ubiquitously expressed in normal mesenchymal cells and its overexpression is frequently associated with increased migratory and invasive capacity of cancer cells [44]. In BORIS-depleted MCF7 cells, an increase of migration capacity was indeed observed and this result could be associated with the up-regulation of *VIMENTIN*. In summary, all these data suggest that BORIS could mediate the regulation of EMT process by transcriptional regulation of *hTERT* and EMT target genes in the non-invasive MCF7 breast tumor cells. More investigations are required to better define the role of BORIS in the EMT process.

The gene expression profiles can give an explanation about the different phenotypes found in the analyzed epithelial tumor cells after BORIS knockdown. Notably, different phenotypes were observed in the two analyzed breast tumor cell lines. MCF7 cells are luminal-like, weakly proliferative and non-invasive, they display epithelial phenotypic markers and express the nuclear hormone receptor (ERα). Instead, MDA-MB-231 cells are basal-like, invasive with ability to migrate, display a mesenchymal phenotype and are ERα-negative. These cell lines are phenotypically and genetically different; therefore, BORIS could affect target genes that are differentially expressed in these cell lines. In addition, MCF7 cells have a different BORIS promoter usage compared to the other analyzed cells. BORIS is transcriptionally controlled by three alternative promoters [45]. The promoters A and C are equally active in MCF7, whereas in the majority of tumor cell lines, BORIS is transcriptionally regulated by the promoter C only. Therefore, in MCF7 and MDA-MB-231, different BORIS proteins could be expressed with potentially diverse biological functions.

To further investigate the biological functions of BORIS in cancer, we induced BORIS expression in BORIS-low expressing epithelial tumor cells. Our observations in MCF7, MDA-MB-231 and HeLa were consistent with those of recent reports in which BORIS overexpression inhibits cell growth [46–48]. Contradictory results showed the relation to the cell cycle progression in BORIS-overexpressing cells. One study demonstrated that BORIS induction did not alter the cell cycle profile of both normal and cancer cell lines [48]. Another study reported that BORIS overexpression in normal HEK293 cells led to an accumulation of cells in S phase, an increase of cell size and a decrease of the cell cycle markers PCNA and Cyclin A [47]. In our experiments, no change of cell size was observed by flow cytometry analysis after BORIS-induction (data not shown). Gene expression profiles showed that BORIS induction promoted *hTERT* expression in MDA-MB-231, HT29 and HeLa cells. These results are in agreement with our previous studies [25, 28]. All together these data further support the evidence that BORIS regulates the *hTERT* gene at the transcriptional level.

The results here presented open new findings to better understand the role of BORIS in tumor development and provide the evidence for a potential new CSC biomarker that could be used as therapeutic target for cancer therapy.

## Supporting Information

S1 Table Primer sequences for qRT-PCR analysis. (PDF)

S1 Fig *BORIS* expression and SP analysis. Representative images of HeLa and HT29 cells after incubation of BORIS-MB and Hoechst 33342. Cells were examined under confocal microscopy, 63X magnification.
(TIFF)
