# Supplementary material for: Different Effects of BORIS/CTCFL on Stemness Gene Expression, Sphere Formation and Cell Survival in Epithelial Cancer Stem Cells
Source: PLoS One. 2015 Jul 17;10(7):e0132977. doi: 10.1371/journal.pone.0132977 (PMC4506091; doi:10.1371/journal.pone.0132977)
Supplement: S1 Table — (PDF) [file pone.0132977.s001.pdf]

Gene	Forward primer	Reverse primer
ECADH	5' TGAAATTGGAAATTTTATTGATGA 3'	5' ATCATAAGGCGGGGCTGT 3'
CK19	5’ CTGCAGATGACTTCCGAACC 3’	5’ TTGATGTCGGCCTCCAC 3’
EpCAM	5’ GCAGCTCAGGAAGAATGTGTC 3’	5’ GACGATTATTATTCACAAAGCAGTTT 3’
NCADH	5’ CCTGAAGCCAACCTTAACTGA 3’	5’ TCTTGGGAACACTATTTCTTCAA 3’
VIMENTIN	5’ CAAAGTGGAATCTTTGCAAGAAG 3’	5’ GCAGCTCCTGGATTTCCTCT 3’
FIBRONECTIN	5’ TAAGCTGTACCATCGCAAACC 3’	5’ CCTCCAGGTGTCACCAATCT 3’
SNAIL	5’ CCCAATCGGAAGCCTAACTA 3’	5’ TAGGGCTGCTGGAAGGTAAA 3’
SLUG	5’ CAGACCCTGGTTGCTTCAA 3’	5’ GCAGTGAGGGCAAGAAAAAG 3’
TWIST	5’ CAGCAGGGCCGGAGAC 3’	5’ CCAGAGTCTCTAGACTGTCCATTTT 3’
