# Supplementary figures and images for: Different Effects of BORIS/CTCFL on Stemness Gene Expression, Sphere Formation and Cell Survival in Epithelial Cancer Stem Cells
Source: PLoS One. 2015 Jul 17;10(7):e0132977. doi: 10.1371/journal.pone.0132977 (PMC4506091; doi:10.1371/journal.pone.0132977)

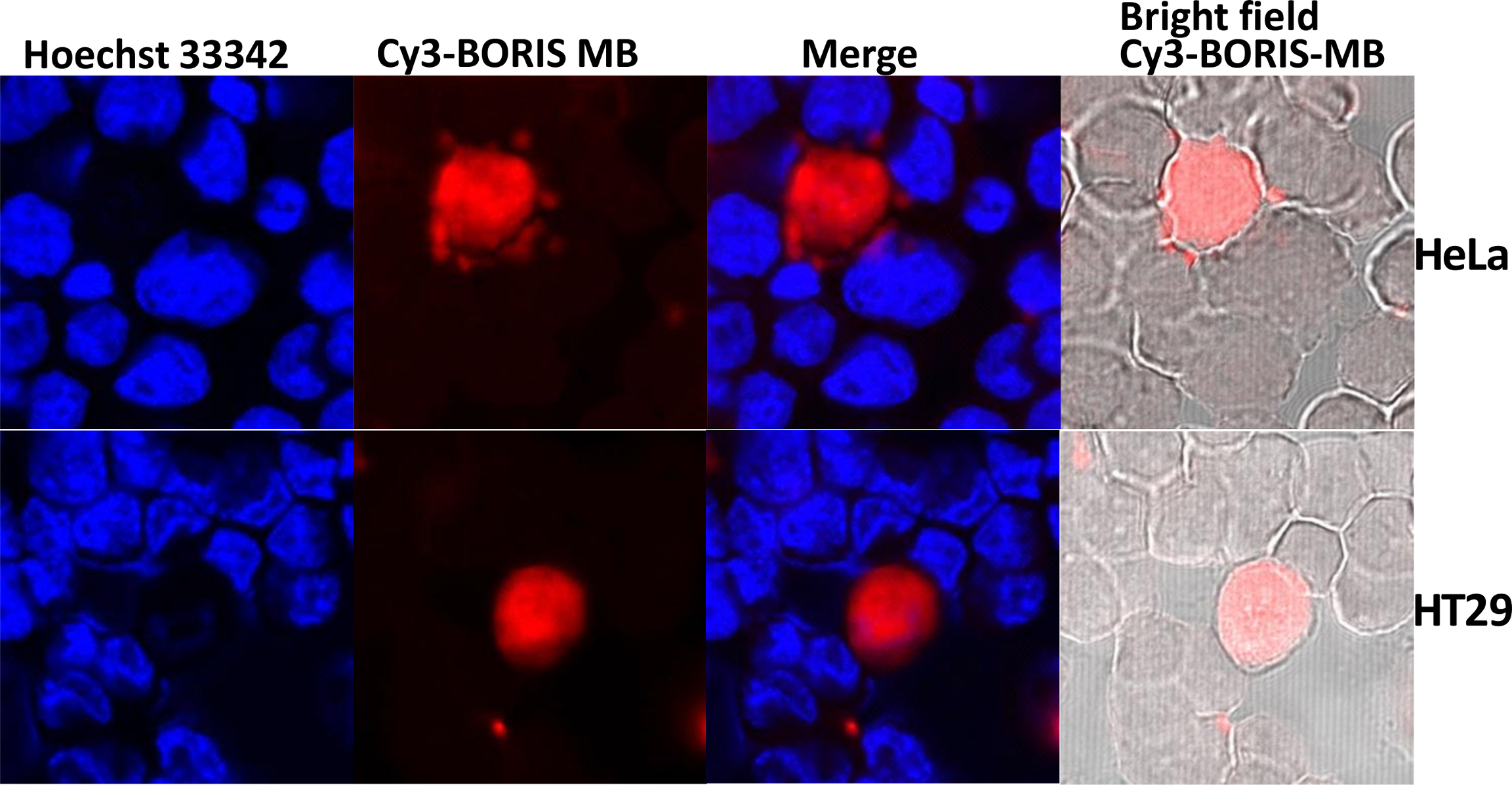

Supplement: S1 Fig — Representative images of HeLa and HT29 cells after incubation of BORIS-MB and Hoechst 33342. Cells were examined under confocal microscopy, 63X magnification. (TIFF) [file pone.0132977.s002.tiff]
